# Cation‐Directed Host–Guest Assembly and Reversible Wheel to Cluster Interconversion in a 20‐Molybdate System

**DOI:** 10.1002/chem.71141

**Published:** 2026-05-22

**Authors:** Arun Pal, Alisher Kuanysh, Vinaya Siby, Jana Hölscher, Nikolai Kuhnert, Albert Masip‐Sánchez, Xavier López, Josep M. Poblet, Maria Boltoeva, Pierre Bauduin, Mohamed Haouas, Emmanuel Cadot, Ulrich Kortz

**Affiliations:** ^1^ School of Science Constructor University Bremen Germany; ^2^ Departament De Química Física i Inorgànica Universitat Rovira i Virgili Tarragona Spain; ^3^ ICSM Univ Montpellier, CEA, CNRS, ENSCM Marcoule France; ^4^ Institut Lavoisier de Versailles Université Paris‐Saclay Versailles France

**Keywords:** DFT, host–guest assembly, polyoxomolybdate wheel, reversible transformation, solution and gas phase studies

## Abstract

We report on the synthesis and structural characterization of the polyoxo‐20‐molybdate wheel, [Mo^VI^_20_O_60_{AsO_2_(CH_3_)_2_}_12_]^12−^ (**Mo_20_**), which can be isolated with an empty cavity (diameter ca. 2 nm) or containing two dimethylarsinate guests, depending on the type of cations used during synthesis. The polyanion **Mo_20_** was characterized by single‐crystal XRD, FT‐IR, and TGA, and in solution by multinuclear NMR (^1^H, ^13^C, ^95^Mo) and ^1^H DOSY, as well as x‐ray scattering (SWAXS) and mass spectrometry (ESI‐MS). We demonstrated a fast, quantitative, and fully reversible transformation of **Mo_20_** to [Mo^VI^_4_O_12_(OH){AsO_2_(CH_3_)_2_}]^2−^ (**Mo_4_**) in aqueous solution, whereas **Mo_20_** is cleanly and quantitatively recrystallized from such solution, indicating a unique phenomenon, which is fully supported by all experimental techniques used as well as DFT and MD calculations.

## Graphical abstract

The polyoxo‐20‐molybdate(VI) wheel [Mo^VI^_20_O_60_{AsO_2_(CH_3_)_2_}_12_]^12−^ (**Mo_20_**) can be prepared with or without a central guest in a controlled, cation‐directed fashion. When **Mo_20_** is dissolved in water, it exhibits an immediate, quantitative, and fully reversible solution transformation to a tetranuclear **Mo_4_** cluster, which transforms in the solid state again to clean **Mo_20_**.

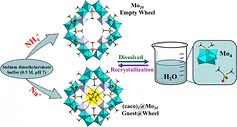

## 1 Introduction

Polyoxometalates (POMs) are notable for their many interesting and varied characteristics, including remarkable self‐assembly abilities, significant redox reactivity, solubility in water and some organic solvents, and extraordinary stability. Owing to their diverse range of properties, POMs are at the forefront of many scientific domains, including catalysis, biomedicine, energy storage, photochemistry, magnetism, and medicinal chemistry [1, 2, 3].

Molybdenum‐based POMs have garnered particular interest due to the discovery of large mixed‐valent species such as the giant {Mo_154_} [4] and {Mo_176_} [5] wheels, {Mo_368_} [6] with a hedgehog‐like shape, and the spherical Keplerate {Mo_132_} [7]. These structures are explored for their potential in supramolecular host‐guest chemistry, drug delivery, magnetism, and materials science [8, 9, 10, 11]. Mixed‐valent molybdates are classified into “blues” and “browns” depending on their molybdenum oxidation states [1, 2, 3]. While polyoxomolybdate blues are more common, browns such as the {Mo_132_} ball remain rare. Functionalizing POMs with covalently attached organic groups can improve solution stability, and various organofunctionalized derivatives using Si, Sn, P, As, and Sb have been documented [12, 13, 14]. Among them, the dimethylarsinate monoanion (CH_3_)_2_As^V^O_2_^−^ (also known as cacodylate) can act as a capping group in both polyoxomolybdate and ‐palladate(II) chemistry [15, 16, 17]. Notably, in 1980 Pope's group reported the tetramolybdodimethylarsinate ion [Mo^VI^_4_O_12_(OH){AsO_2_(CH_3_)_2_}]^2−^ (**Mo_4_**) [18]. In 2022, we reported the dimethylarsinate‐capped mixed‐valent polyoxo‐30‐molybdate wheel [Mo^VI^_18_Mo^V^_12_O_84_{AsO_2_(CH_3_)_2_}_18_]^18−^ and the neutral tetramolybdenum(V)‐oxo cluster [Mo^V^_4_O_8_{AsO_2_(CH_3_)_2_}_4_] [19]. Last year, we reported a series of fully reduced organoarsonate‐/organophosphonate‐ and dimethylarsinate‐functionalized hexamolybdates(V) [20, 21]. We have demonstrated that the use of dimethylarsinate as a hetero group enables the construction of Mo‐oxo frameworks at near‐neutral pH, which significantly improves the solution stability under physiological conditions.

Here, we report on the synthesis of a polyoxo‐20‐molybdate(VI) wheel and its exciting solution and gas phase properties.

## 2 Results and Discussion

**FIGURE 1:**
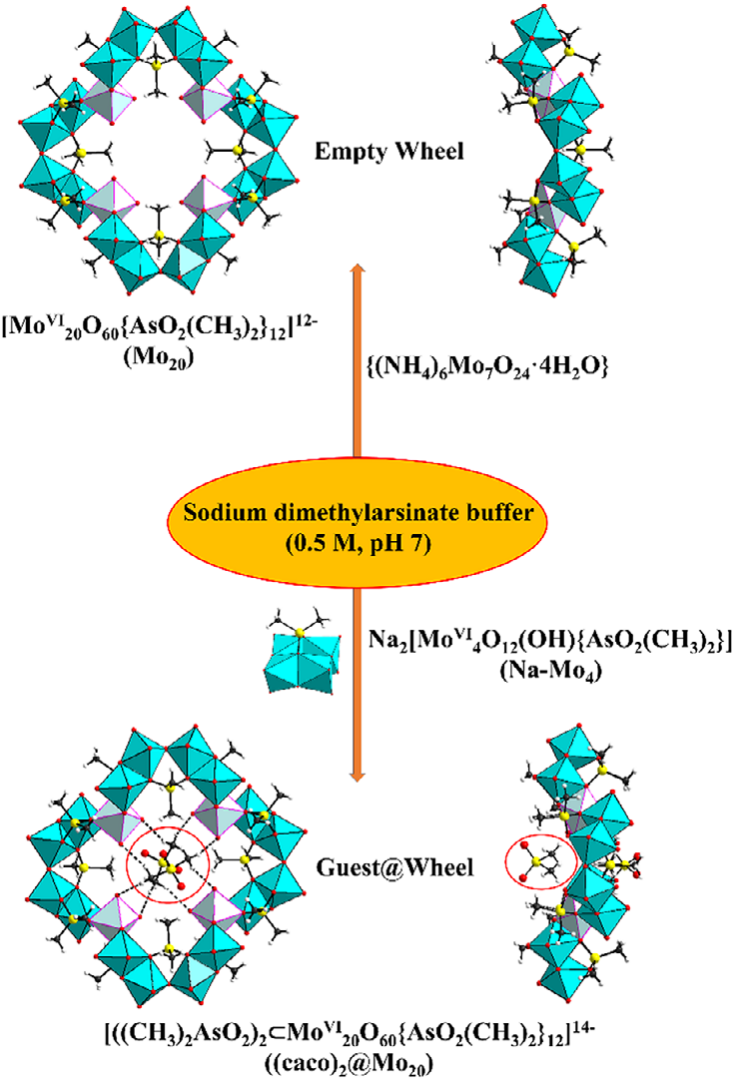
Structural representation of the empty **Mo_20_** polyanion in top and side view (upper) and the dimethylarsinate guest ion‐containing analogue **(caco)_2_@Mo_20_** (lower). Color code: MoO_6_, cyan polyhedra; O, red; As, yellow; C, black; H, white.

The polyanion [Mo^VI^_20_O_60_{AsO_2_(CH_3_)_2_}_12_]^12−^ (**Mo_20_**) was synthesized by reacting ammonium heptamolybdate tetrahydrate with sodium dimethylarsinate solution (0.5 M) of pH 7.0 using heating conditions at ∼80°C for 1 h (see synthetic procedure in Supporting Information). Single‐crystal x‐ray analysis shows that the polyanion is crystallized as a mixed sodium‐ammonium salt in the orthorhombic system with space group *Pnma* (see Table S1) and displays a wheel‐shaped structure. The polyanion **Mo_20_** comprises four {Mo^VI^_4_As_3_} fragments that are connected alternatively by four octahedral {*o*‐Mo^VI^_1_} units in a cyclic fashion, resulting in a wheel with a diameter of ca. 2.0 nm (see Figure 1 (upper), Figures S4 and S5). The neighbouring {Mo^VI^_4_As_3_} groups are oriented in opposite directions, while the {*o*‐Mo^VI^_1_} are all positioned in the same plane, resulting in idealized *D*_2_ *_d_* point group symmetry of **Mo_20_**. Concerning the newly emerged {Mo^VI^_4_As_3_}, two pairs of edge‐sharing octahedra are linked by a corner‐shared oxygen atom, with the surface being decorated by three dimethylarsinate groups on both sides. To complete the ring, the {*o*‐Mo^VI^_1_} units adopt the edge‐sharing mode to bridge the {Mo^VI^_4_As_3_} fragments. A closer inspection of **Mo_20_** revealed that both hydrophilic (oxygens from {*o*‐Mo^VI^_1_}) and hydrophobic groups (methyl from dimethylarsinate) point toward the wheel center, signifying the hollow region (diameter of ca. 0.8 nm) might be suitable for encapsulating guest molecules.

Considering the presence of a central cavity in **Mo_20_**, we made much effort to try to fill it with a guest molecule or ion. We could isolate a host‐guest‐type derivative with two dimethylarsinate ion guests inside **Mo_20_**, namely [((CH_3_)_2_AsO_2_)_2_⊂Mo^VI^_20_O_60_{AsO_2_(CH_3_)_2_}_12_]^14−^ (**(caco)_2_@Mo_20_**), see Figure 1 (lower), which was prepared by dissolving the sodium salt of **Mo_4_** in sodium dimethylarsinate buffer (0.5 M, pH 7) or alternatively, in an aqueous solution of **Na‐Mo_4_** (0.02 mmol) and sodium dimethylarsinate (0.1 mmol) (solution pH∼6), see Figure S6 (see synthetic procedure in Supporting Information). In **(caco)_2_@Mo_20_**, two dimethylarsinate ions occupy the cavity of the **Mo_20_** wheel (acting as wheel‐caps), with their hydrophobic methyl groups pointing to the center of the wheel and their hydrophilic oxo groups pointing away from it (Figure 1, lower). The dimethylarsinate guests are stabilized by supramolecular forces (mainly C‐H···O interactions), for details see Figure 1 (lower) and Figure S7. It is important to note that the absence of ammonium ions appears to be the key for preparing the host‐guest assembly, indicating an important cation‐directed effect toward the formation of a host‐guest assembly.

It was also possible to recrystallize **Mo_20_** from an NMR tube, and single‐crystal XRD studies on this material (abbreviated as **Na‐NH_4_‐Mo_20_‐rec**) showed the presence of **Mo_20_**, demonstrating the solution stability of this polyanion (see Table S1 and Figure S8).

Bond valence sum (BVS) calculations on **Na‐NH_4_‐Mo_20_** and **Na‐NH_4_‐Mo_20_‐rec** demonstrated a +6 oxidation state for all Mo atoms. The thermogram of **Na‐NH_4_‐Mo_20_** displays ∼14.8% weight loss until 140 °C, representing the loss of 40 (13.1%) water molecules, and then remains stable up to 230 °C. The polyanion structure degrades slowly upon further heating (Figure S9). The thermogram of **Na‐Mo_20_** displays ∼16.6% weight loss until 140 °C, representing the loss of 56 (16.5%) water molecules, and then remains stable up to 280°C (Figure S10).

### 2.1 Solution NMR Studies

The ^1^H‐NMR spectrum of an aqueous solution of **Na‐NH_4_‐Mo_20_** exhibits two signals at 1.80 and 2.29 ppm (Figure S11). The signal at 1.80 ppm corresponds to free dimethylarsinate (CH_3_)_2_AsO_2_^−^ ions, which was confirmed by adding free (CH_3_)_2_AsO_2_H to the solution (Figure S13). Similarly, the ^13^C NMR spectrum shows two signals at 17.7 and 20.7 ppm (Figure S12) and, in complete agreement with ^1^H NMR, the signal at 17.7 ppm corresponds to free dimethylarsinate ions (Figure S14). These observations suggest that in aqueous solution, **Mo_20_** is unstable and transforms to another polyanion with partial release of dimethylarsinate capping groups. We identified this transformation product as Pope's polyanion [Mo_4_O_12_(OH){AsO_2_(CH_3_)_2_}]^2−^ (**Mo_4_**), which was reported in 1980 [18]. The transformation of **Mo_20_** to **Mo_4_** in solution can be rationalized by equation (3) in the Supporting Information.

**FIGURE 2:**
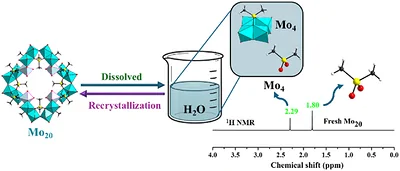
Clean structural transformation of **Mo_20_** to **Mo_4_** upon dissolution in water and complete reversibility upon crystallization, as shown by solution ^1^H NMR and single‐crystal XRD. See text for more details. Color code: MoO_6_, cyan polyhedra; O, red; As, yellow; C, black; H, white.

Indeed, the integration ratios of the ^1^H NMR signals for 1.80 and 2.29 ppm are about 3:2, which corresponds roughly to the 7 equivalents of dimethylarsinate and the 5 equivalents of **Mo_4_** formed from every one equivalent of **Mo_20_**. As a control, we measured the ^1^H and ^13^C NMR spectra of neat **Mo_4_** dissolved in water and observed a singlet at 2.29 ppm (^1^H) and one at 20.7 ppm (^13^C), see Figures S13 and S14, thereby confirming our assignments. Also, we observed the formation of crystals in the NMR tube in which **Mo_20_** had originally been dissolved, and we conducted single‐crystal XRD, which showed the presence of **Mo_20_** (Figure S8), also confirmed by FT‐IR (Figure S2). This observation suggests that when **Mo_20_** is dissolved in water, it cleanly and instantaneously transforms to **Mo_4_**, which then crystallizes out again cleanly as **Mo_20_** (Figure 2, Figure S15). The ^1^H and ^13^C NMR spectra of the guest‐centered **(caco)_2_@Mo_20_** dissolved in water show exactly the same signals as the empty **Mo_20_** (Figures S16 and S17), suggesting that the host‐guest assembly is not more stable than the empty wheel in aqueous solution.

**FIGURE 3:**
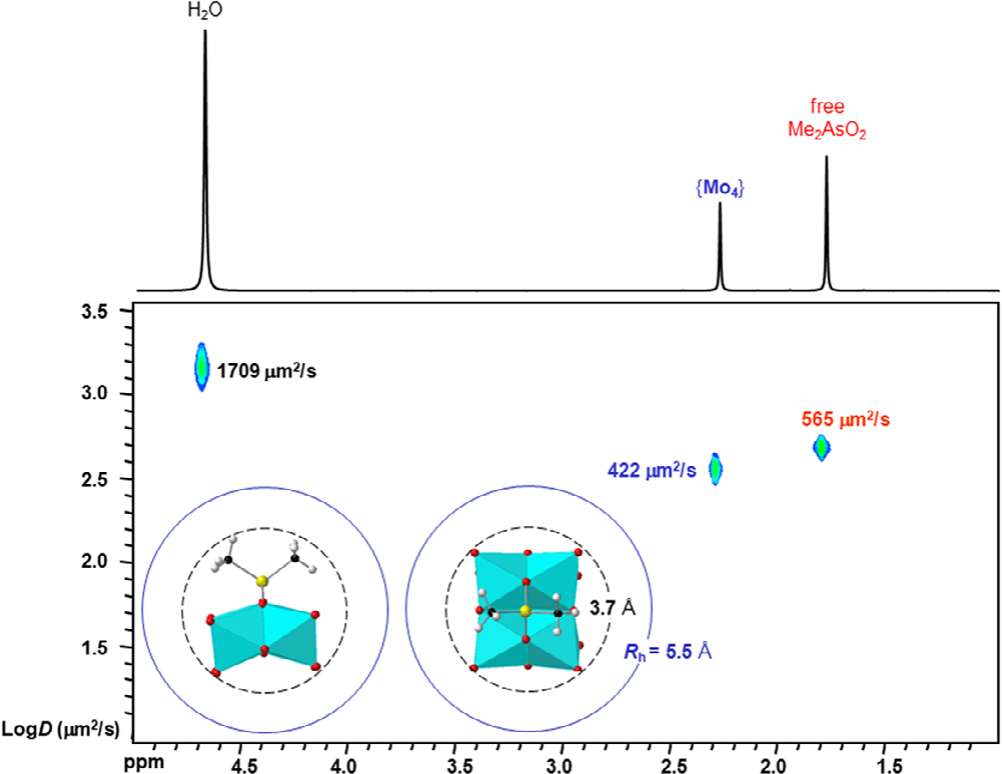
^1^H DOSY NMR of a 23 mM aqueous solution of **Na‐NH_4_‐Mo_20_** acquired at 23°C. Inset: Two views of the **Mo_4_** ion fitting the hydrodynamic radii calculated with 422 µm^2^/s.

**FIGURE 4:**
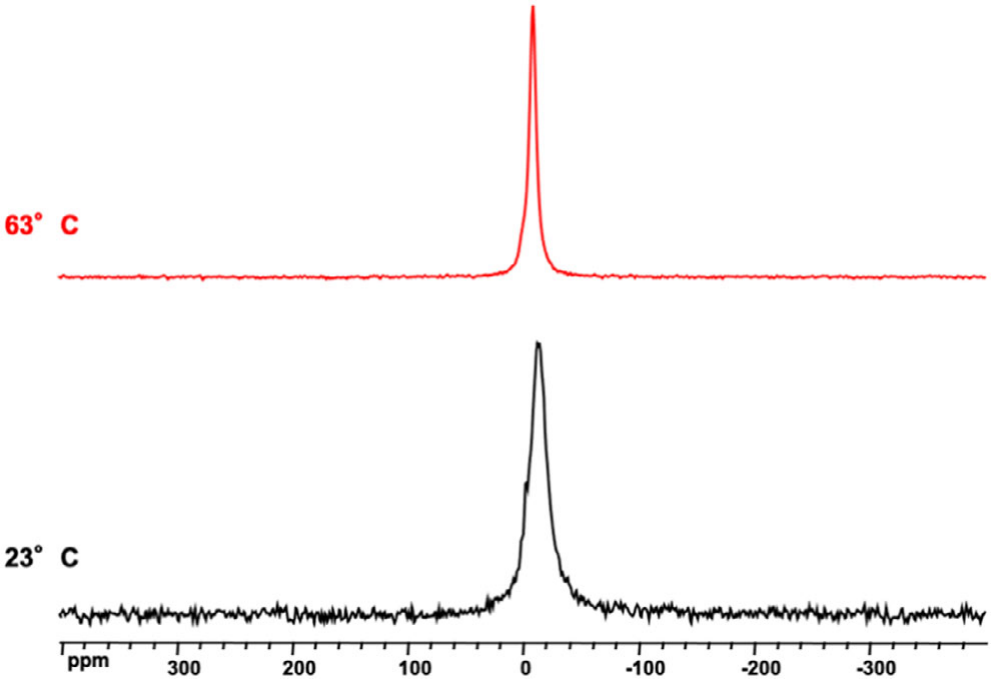
^95^Mo NMR of a 23 mM aqueous solution of **Na‐NH_4_‐Mo_20_** acquired at 23 and 63°C. Note the narrowing of the signal with increasing temperature.

Further evidence for the transformation of **Mo_20_** to **Mo_4_** in solution can be obtained from ^1^H DOSY and ^95^Mo NMR. The DOSY NMR spectrum of a 23 mM solution of **Na‐NH_4_‐Mo_20_** in D_2_O (Figure 3) indicates diffusion coefficients *D* = 565 and 422 µm^2^/s for the signals at 1.80 and 2.29 ppm, respectively. From these values, using the Stokes‐Einstein relation ($D\;=\frac{k_{B}T}{6\pi \eta R_{h}}$) approximate hydrodynamic radii *R*_h_ of about 4.1 and 5.5 Å, respectively, can be derived, which reasonably correspond to the molecular size of solvated free dimethylarsinate and the **Mo_4_** polyanion. *k*_B_ is the Boltzmann constant, *T* is the absolute temperature, and *η* the solvent viscosity. The measured values can in no way correspond to the **Mo_20_** polyanion with a diameter of ca. 2.0 nm. On the other hand, ^95^Mo NMR of the same solution (Figure 4) shows a single relatively narrow signal (*δ* = −8.5 ppm, LW = 170 Hz) at 63 °C, indicating a unique molybdenum environment as is present in the small and compact **Mo_4_** polyanion. On the other hand, the larger, wheel‐shaped polyanion **Mo_20_** is expected to exhibit three distinct Mo sites.

### 2.2 ESI Mass Spectrometry

**FIGURE 5:**
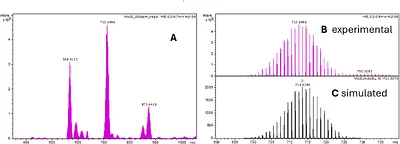
ESI‐mass spectrum in negative ion mode of **Na‐NH_4_‐Mo_20_** in aqueous solution; (A) full scan spectrum; (B) experimental spectrum zoomed in around *m/z* 712 of [Mo_4_C_2_H_6_AsO_14_]^−^; (C) simulated spectrum zoomed in around *m/z* 712 of [Mo_4_C_2_H_6_AsO_14_]^−^.

The ESI mass spectrum in negative mode of **Na‐NH_4_‐Mo_20_** in aqueous solution shows three major groups of signals centred around *m/z* = 588, 712, and 872, with *m/z* = 712 as the base peak. All isotope envelopes could be assigned to singly charged ions with isotope patterns corresponding to typical **Mo_4_** species. The major species centred around *m/z* = 712 could be assigned to an ion with a molecular formula of [Mo_4_C_2_H_6_AsO_14_]^−^ corresponding to **Mo_4_** with the loss of a hydroxide ion, yielding a singly charged anion. The simulated and experimental spectra are in excellent agreement, as shown in Figure 5. The ions centred around *m/z* = 588 and 872 could be assigned to a **Mo_4_** lithium ion adduct species with loss of a dimethylarsinate group or addition of a dimethylarsinate group, respectively (Figure S18), again with simulated and experimental spectra in excellent agreement. Smaller minor peaks could be assigned to sodium and potassium ion adducts, respectively, with alkali ions probably leaching out from the glassware used for sample preparation.

The ESI mass spectra hence clearly confirm the hypothesis from solution NMR data, demonstrating a clean transformation of a **Mo_20_** wheel to five **Mo_4_** ions accompanied by loss of seven dimethylarsinate ligands.

### 2.3 Small‐ and Wide‐Angle X‐ray Scattering (SWAXS)

Small‐ and wide‐angle x‐ray scattering (SWAXS) was employed to investigate the solution behavior of **Mo_20_** in water, as this technique is a method of choice for studying polyoxometalate species and their interactions in solution [22]. Aqueous solutions were prepared by dissolving **Na‐NH_4_‐Mo_20_** crystals in water so as to obtain a stock solution of 30 mM, which was subsequently diluted to 5 mM. Molar concentrations given here were calculated based on the **Mo_20_** polyanion.

**FIGURE 6:**
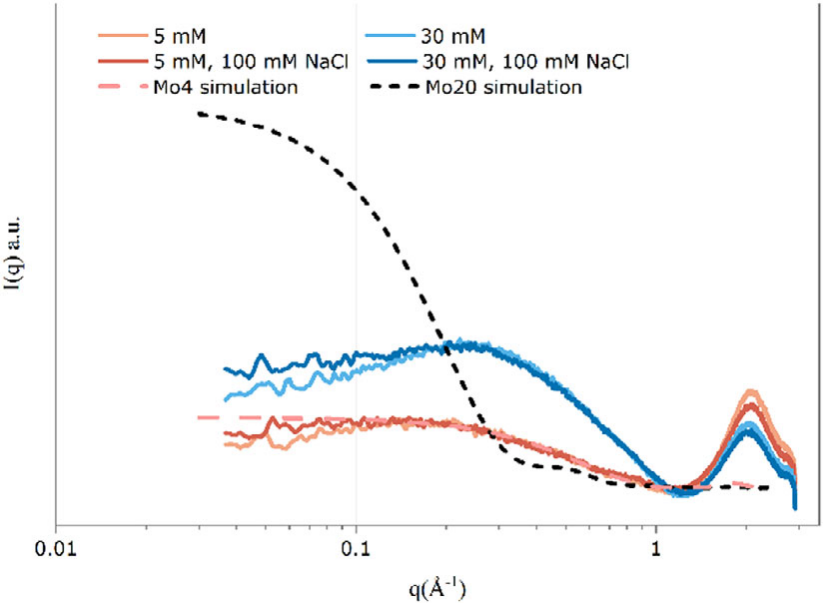
SWAXS profiles of aqueous solutions prepared from dissolved **Na‐NH_4_‐Mo_20_** crystals at 5 and 30 mM concentrations. The curves exhibit features characteristic of compact nanoscale species, with concentration‐dependent variations indicating interparticle repulsions. The addition of 100 mM NaCl suppresses the low‐q intensity drop, suggesting electrostatic origins of these interactions. Simulated scattering patterns based on the crystal structures of **Mo_20_** and **Mo_4_** are included for comparison.

The corresponding scattering profiles are shown in Figure 6. At large q (wave vectors around 2 Å^−1^), only the broad water structure factor peak is observed, indicating the absence of any significant structural features within this q‐range and concentration domain. At lower q values (below 1 Å^−1^), the scattering intensity exhibits a clear upturn, consistent with the form factor of small compact objects with a size close to one nanometer.

A slight decrease in scattered intensity is observed at small q (below around 0.2 Å^−1^) for the 5 mM solution, which becomes more pronounced at 30 mM. This behavior is characteristic of repulsive interactions between scattering objects, see for example [23] showing strong electrostatic repulsions between Keggin polyanions. To elucidate the origin of these interactions, solutions were prepared in the presence of 100 mM NaCl. In this case, the downturn in the low‐q intensity completely vanished, suggesting that the repulsive interactions are electrostatic in nature. This interpretation is consistent with the fact that objects in solution are charged species with dissociated counterions, as observed for classical polyoxometalates in aqueous media.

The scattering curves of **Mo_20_** and **Mo_4_** were simulated using the atomic coordinates obtained from their crystallographic structures, using the CRYSOL software, which employs a multipole expansion of the scattering amplitudes to compute the spherically averaged intensity profile [24]. The simulated curve for **Mo_20_** shows an excess of scattering intensity at low q values (below approximately 0.5 Å^−1^), which is not observed experimentally. In contrast, the simulated profile of the **Mo_4_** species is in very good agreement with the experimental scattering data (see the 5 mM spectrum). This consistency strongly supports the conclusion that only **Mo_4_** is in solution, having been generated by rapid and quantitative transformation of solid **Mo_20_** after dissolution in water, which is in full agreement with the solution NMR and ESI‐MS results (vide supra).

Furthermore, **Mo_20_** (which we know transforms to **Mo_4_** in aqueous solution, as evidenced by NMR, ESI‐MS, and SWAXS) was also investigated for its potential superchaotropic character, a property observed in inorganic, nanometer‐sized charged species when their charge density typically falls below ∼12 charges/nm^3^, leading to a propensity to bind to hydrophilic, hydrated molecules or surfaces [22, 25]. The cloud point of C8E4 was measured upon addition of **Na‐NH_4_‐Mo_20_** (up to 30 mM), following a previously reported procedure [26], in order to assess this behavior. No change in the cloud point was observed, as expected given the relatively high charge density of **Mo_20_** (∼17 charges/nm^3^) and **Mo_4_** (∼14.3 charges/nm^3^), which is above the superchaotropic threshold. It should be stressed that the charge density values discussed here were estimated by considering only the polyanion core, without including the capping groups in the calculation of the molecular volume.

It should also be emphasized that previously studied POMs based on W or Mo were typically Keggin or Dawson‐type structures, formed without (metal‐)organic capping groups such as the dimethylarsinate moiety used here. Such capping groups may introduce additional effects, for example, a slight hydrophobic character that could, in principle, influence interfacial or solution behavior. For this reason, the cloud point experiment was performed to probe the overall solution properties of the compound, even though we did not expect it to display superchaotropic behaviour based solely on its high charge density, which is above the values typically associated with superchaotropic nanometric ions. The cloud point measurement thus also served to assess whether any hydrophobic contribution from the capping group might manifest under these conditions, which was not the case.

### 2.4 Computational Results

To support some aspects of the experimental observations, we combined density functional theory (DFT) with atomistic classical molecular dynamics (MD) simulations on the target systems. MD calculations deliver a detailed and time‐averaged picture of the spatial organization of counterions in the solution, notably whether ion‐pairing with the complexes occurs. DFT calculations were carried out on those MD configurations, reproducing and helping to rationalize the ^1^H NMR chemical shifts of the species in solution. In addition, they furnish a quantitative thermodynamic description of the probable hydrolysis steps driving the transformation of **Mo_20_** to **Mo_4_**.

#### 2.4.1 Thermodynamics of the Mo_20_ Hydrolysis

Although the full hydrolysis/crystallization mechanism is too complex to model explicitly, we elucidated whether the hydrolysis of **Mo_20_** is thermodynamically favourable. To this end, all species in Reaction 3 (in Supporting Information) were evaluated by DFT. At pH 7 (a_H+_ = 10^−7^), complete hydrolysis is strongly exergonic (Δ*G* = −62.3 kcal mol^−1^), accounting for the experimental absence of **Mo_20_**. The driving force arises from dispersing the −12 charge of a single polyanion into several smaller anions, substantially increasing solvation stabilization, together with the standard entropic gain from generating more particles. Despite the simplicity of the model and the well‐known challenges in treating highly charged polyanions and their counterions within continuum solvation methods, the result is robust. For more information, see the Computational Details in Supporting Information.

#### 2.4.2 Ion Pairing and Solvation of Mo_20_ and Mo_4_

**FIGURE 7:**
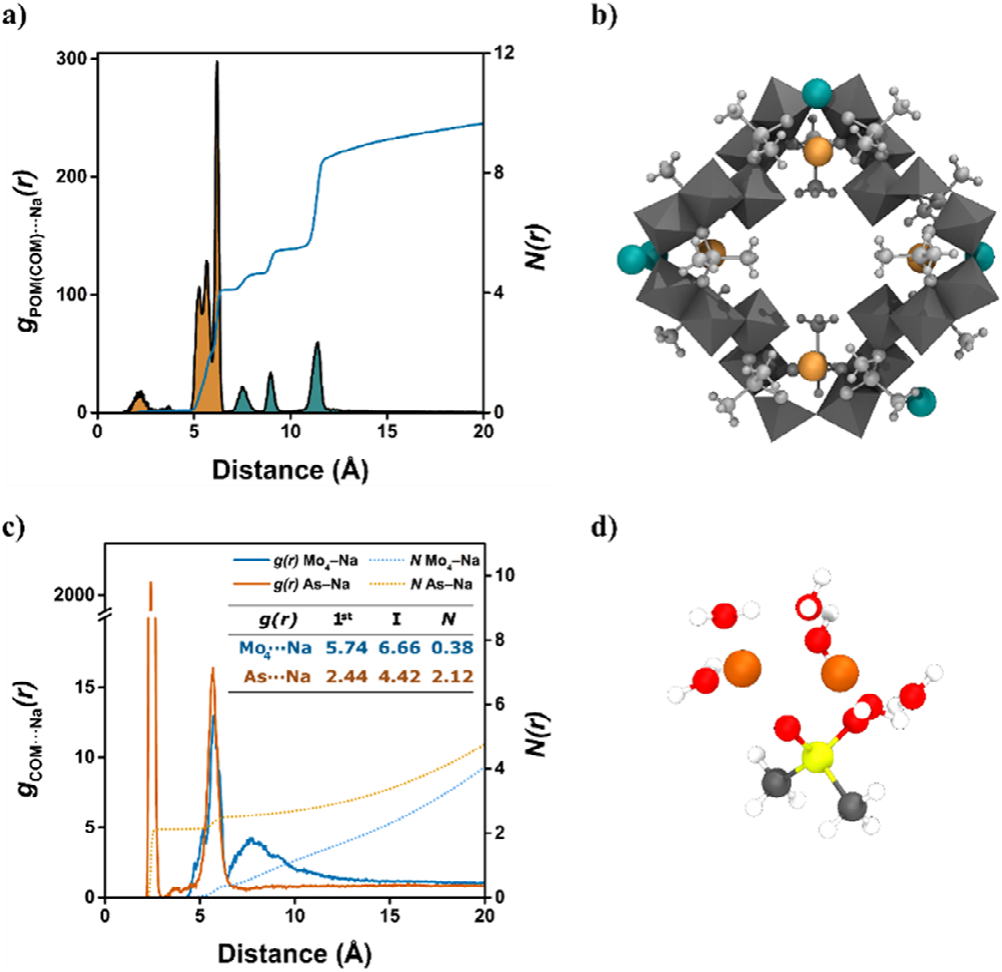
Classical Molecular Dynamics simulation. (a) Radial distribution function (RDF, black line) for **Mo_20_** ···Na^+^ using the POM center of mass (COM) and the Na center as references, together with its coordination integral (blue line). Peaks are color‐coded to distinguish Na^+^ ions located inside the POM cavity (orange) from those outside (green). (b) Representative snapshot of **Na‐Mo_20_** showing 9 Na^+^ cations ion‐paired to the metal‐oxide. The polyanionic scaffold is rendered in grayscale; Na^+^ ions retain the color scheme from panel (a): orange for internal, green for external. (c) Combined RDFs for **Mo_4_** ···Na^+^ (solid blue line) and dimethylarsinate···Na^+^ (solid orange line), referenced to the respective COMs and Na nuclei, with their integrations (blue and orange dashed lines). The first‐peak position (first), integration cutoff (*I*), both in Å, and the corresponding coordination number (*N*) are reported. (d) Representative snapshot of Na_2_(H_2_O)_6_‐dimethylarsinate extracted from the MD trajectory and used for the δ(^1^H) calculations. All data were sampled every 1 ps over the final 25 ns of the trajectory.

The MD results (Figure 7) for **Na‐Mo_20_** in solution show that the alkali cations form very strong ion pairs with **Mo_20_**, organizing into two groups: four long‐lived Na^+^ ions residing within the polyanion cavity, evidenced by sharp peaks in the RDF (Figure 7a, orange‐shaded region), and four to five Na^+^ ions placed at the outer surface of **Mo_20_** (*N* = 8.58), exposed to solution, reflected in the broad, low‐intensity peaks colored green in Figure 7a. Assuming that the **Mo_20_** anion remains intact in the aqueous phase and does not dissociate into four **Mo_4_** units, we analyzed the preferred cation positions around the molecular oxide and obtained a representation (Figure 7b) that is consistent with the **Na‐Mo_20_** arrangement observed in the crystal (Figure S6). Moreover, the simulations reveal that in the presence of NH_4_^+^ cations, these preferentially interact with **Mo_20_**, occupying three of the four internal sites and leaving the fourth one to two Na^+^, as seen in Figures S19 and S20.

Separately, we simulated the behaviour of **Mo_4_** in solution with a box containing five **Mo_4_** units, seven dimethylarsinate anions ([Me_2_AsO_2_]^−^), and 17 Na^+^ ions in water. Strikingly, as shown by the RDF in Figure 7c, essentially no cations (ca. ≤1) associate with **Mo_4_**. Instead, most alkali (*N* = 2.1) form strong, long‐lived ion pairs with dimethylarsinate (Figure 7d). Upon adding ammonium, the cation‐dimethylarsinate ion pairs share, on average, *N* = 0.93 Na^+^ and *N* = 1.52 NH_4_^+^ per [Me_2_AsO_2_]^−^ (Figures S19 and S20). Likewise, **Mo_4_** exhibits negligible cation association (Figures S19 and S20), as its water solvation shell shields this structure from direct cation contact.

To align the dynamics simulated with the experimental data, we computed self‐diffusion coefficients (*D*) for **Mo_20_**, **Mo_4_**, and [Me_2_AsO_2_]^−^ from the long‐time slope of the mean‐squared displacement (Einstein relation; see Computational Details in Supporting Information for more information), applying standard finite‐size corrections (Yeh‐Hummer; see Computational Details in Supporting Information). The *D* values obtained are compared directly with the ^1^H DOSY measurements in Figure 3, showing excellent agreement: 433 µm^2^ s^−1^ (calc.) vs 422 µm^2^ s^−1^ (exp.) for **Mo_4_**, and 594 µm^2^ s^−1^ (calc.) vs 565 µm^2^ s^−1^ (exp.) for free dimethylarsinate. This concordance already indicates that, in solution, **Mo_20_** dissociates into **Mo_4_** plus dimethylarsinate. Consistently, if **Mo_20_** were present in solution, its predicted diffusivity would be substantially lower, estimated *D* ≈ 153 µm^2^ s^−1^.

**TABLE 1:** Computed and experimental (in parentheses) ^1^H NMR chemical shifts (in ppm) and relative abundance (%) of the species in solution.

Ion	δ_calc._ (δ_exp._)	%_calc._ (%_exp._)
**Mo_4_**	2.36 (2.29)	42 (40)
Free (CH_3_)_2_AsO_2_^−^	1.74 (1.8)	58 (60)
**Mo_20_**	2.33–2.70 (–)	100% (–)

#### 2.4.3 Calculation of Accurate δ(^1^H) of Species in Solution

Building on the solution‐phase behavior of the species described above, we next connect structure to spectroscopy. We selected representative snapshots from MD trajectories that capture either **Na‐Mo_20_** or Na‐dimethylarsinate ion pairing and computed the corresponding ^1^H chemical shifts. For **Mo_4_**, given its negligible cation association, we treated it as an isolated, solvated species. We deliberately focus on ^1^H rather than ^13^C: in organo‐arsinates, the ^13^C resonances are strongly affected by heavy‐atom‐to‐light‐atom (HALA) effects [27], which are highly sensitive to spin–orbit/relativistic contributions and local anisotropy, thereby complicating both calculation and interpretation beyond the scope of this study.

Table 1 compiles the computed ^1^H NMR chemical shifts. The agreement with experiment is excellent, with a mean deviation of ±0.06 ppm. Consequently, the inter‐peak separation is also captured: 0.49 ppm experimentally versus 0.62 ppm computationally. Moreover, the relative ^1^H integrals agree with the theoretical proton ratios (and ensemble populations), further supporting the assignments. Notably, reproducing the ^1^H shifts of free dimethylarsinate requires explicit inclusion of the cations that form strong ion pairs with the anion; without these counterions, the shifts collapse to 1.24 ppm, far from the experimental 1.80 ppm. Another advantage of the calculations is that they allow us to probe species that are not necessarily stable in solution, such as **Na‐Mo_20_**. If **Mo_20_** were present in solution, its ^1^H shifts would be expected in the range 2.3–2.7 ppm. While one of the dimethylarsinate peaks bound to **Mo_4_** overlaps with the dimethylarsinate‐**Mo_20_** region, the absence of additional, more downfield signals supports the conclusion that **Mo_20_** is not experimentally stable in solution.

## 3 Conclusion

We have discovered an important cation‐directed effect in the synthesis of a host‐guest assembly. In a sodium and ammonium ion‐containing aqueous medium, the 20‐molybdate wheel **Mo_20_** is formed with an empty cavity, but under sodium‐only conditions, the central cavity is filled by two dimethylarsinate ((CH_3_)_2_AsO_2_^−^) guest ions, resulting in **(caco)_2_@Mo_20_**. The orientation of the guests with the two methyl groups pointing into the cavity and the oxo groups pointing away reflects the fact that the cavity is more hydrophobic than the rest of the wheel, due to the orientation of the 12 dimethylarsinate groups, which are an integral part of **Mo_20_**. It is likely that other amphiphilic guests can be bound, rendering the **Mo_20_** potentially interesting as a drug carrier. However, in order to achieve this, the solution stability of **Mo_20_** must be increased, possibly by using guests with longer hydrophobic and/or hydrophilic functionalities. We have observed that when the solid salts of **Mo_20_** or **(caco)_2_@Mo_20_** are added to water, they both transform instantaneously and cleanly to [Mo^VI^_4_O_12_(OH){AsO_2_(CH_3_)_2_}]^2−^ (**Mo_4_**), accompanied by the release of dimethylarsinate ions. This phenomenon has been investigated by various experimental solutions and gas phase techniques and was rationalized by theoretical calculations. Interestingly, when a solution of **Mo_4_** is left standing for crystallization, only clean **Mo_20_** is formed in the solid state. Such a solid‐state to solution and back to solid‐state transformation of two species is unprecedented to the best of our knowledge. Our work to date is an inspiration to investigate polyoxometalate wheel‐based host‐guest chemistry further.

## Conflicts of Interest

The authors declare no conflicts of interest.

## Supporting information



Experimental section, physical measurements, details of ESI mass spectrometry, small and wide‐angle x‐ray scattering (SWAXS), cloud point determination, synthetic procedures, x‐ray crystallography, crystallographic data, POM structures, NMR spectra, FT‐IR spectra, thermogram, ESI‐MS, and computational details, MD/DFT plots (PDF). CCDC 2517434 (**Na‐NH_4_‐Mo_20_**), 2517435 (**Na‐NH_4_‐Mo_20_‐rec**) and 2517436 (**Na‐Mo_20_**). The authors have cited additional references within the Supporting Information [18, 28, 29, 30, 31, 32, 33, 34, 35, 36, 37, 38, 39, 40, 41, 42, 43, 44, 45].
