# Supplementary material for: Cation‐Directed Host–Guest Assembly and Reversible Wheel to Cluster Interconversion in a 20‐Molybdate System
Source: Chemistry. 2026 May 22;32(33):e71141. doi: 10.1002/chem.71141 (PMC13532167; doi:10.1002/chem.71141)
Supplement: Supplementary file 1 [file CHEM-32-e71141-s001.docx]

**Supporting Information**

**Cation-Directed Host–Guest Assembly and Reversible Wheel to Cluster Interconversion in a 20-Molybdate System**

Arun Pal,^a^ Alisher Kuanysh,^a^ Vinaya Siby,^a^ Jana Hölscher,^a^ Nikolai Kuhnert,^a^ Albert Masip-Sánchez,^b^ Xavier López,^b^ Josep M. Poblet,^b^ Maria Boltoeva,^c^ Pierre Bauduin,^c^ Mohamed Haouas,^d^ Emmanuel Cadot,^d^ and Ulrich Kortz*^a^

*^a^School of Science, Constructor University, Campus Ring 1, 28759 Bremen, Germany,*

Email: [ukortz@constructor.university](mailto:ukortz@constructor.university)

*^b^Departament de Química Física i Inorgànica, Universitat Rovira i Virgili, Tarragona 43007, Spain*

*^c^ICSM, Univ Montpellier, CEA, CNRS, ENSCM, Marcoule, France*

*^d^Institut Lavoisier de Versailles, Université Paris-Saclay, Versailles 78035, France*

**Experimental Section**

**Materials and reagents.** All commercially available chemicals were received as reagent-grade quality, with no further purification before use. The sodium salt of tetramolybdodimethylarsinate **Mo_4_** was synthesized using the reported procedure.^[1]^

**Physical Measurements.** All solution NMR spectra were measured in D_2_O. ^1^H-NMR, ^13^C-NMR, and ^95^Mo-NMR spectra were recorded on a Bruker Avance 400 spectrometer at Larmor frequencies of 400.1, 100.6, and 26.1 MHz, respectively, using standard 5 mm NMR tubes. The 1D ^1^H spectra were recorded with one pulse sequence at 30° flip angle (pulse duration 3.0 μs), using 1 s recycle delay, 1.6 s acquisition time, and 16 number of scans. Translational diffusion measurements were performed at 23 °C using Bruker's “ledbpgs2s” stimulated echo DOSY pulse sequence including bipolar and spoil gradients. Apparent diffusion coefficients were obtained using an adapted algorithm based on the inverse Laplace transform stabilized by maximum entropy. The ^13^C spectra were recorded under proton decoupling, using 2.5 s recycle delay, 0.6 s acquisition time, and ca. 8k number of scans. The ^95^Mo spectra were recorded at 63 °C using Hahn-echo sequence with 80 ms echo delay, and 0.1 s recycle delay, 0.02 s acquisition time, and ca. 1024k number of scans. Additional ^1^H and ^13^C NMR spectra were performed on a JEOL ECX 400 MHz instrument. Chemical shifts are scaled with respect to standards (δ = 0 ppm): Me_4_Si (1% CDCl_3_) for ^1^H and ^13^C, and Na_2_MoO_4_ (D_2_O, 2 M) for ^95^Mo. The FT-IR spectra were acquired on a Nicolet-Avatar 370 spectrometer from 4000 to 400 cm^−1^ using KBr disks. Thermogravimetric analyses (TGA) were performed on a TA Instruments Q600 apparatus, using a nitrogen gas flow and heating rate of 5 °C per minute from room temperature to 500 °C. Elemental analyses were performed by Analytische Laboratorien in Lindlar, Germany.

**ESI mass spectrometry.** High-resolution mass spectra were recorded using a Bruker Daltonics QTOF Impact HD mass spectrometer employing both negative and positive electrospray ionization modes. The QTOF Impact mass spectrometer (Bruker Daltonics) was fitted with an ESI source and external calibration was achieved with 10 mL of 0.1 M sodium formate solution. Instrument ion source and tubing were rinsed with methanol. Calibration was carried out using the enhanced quadratic calibration mode. All MS measurements were performed in both negative and positive ion modes. Samples were measured as direct infusions at a concentration of 10 µg/mL in deionized water at a flow rate of 180 µL/min. Samples were prepared by dissolving 1 mg of POM salt in 1 mL of deionized water followed by a 1:100 dilution. Spectral simulations were carried out in Data Analysis 4.1 (Bruker Daltonics, Bremen).

**Small and wide angle X-ray scattering (SWAXS).** SWAXS measurements using Mo radiation (λ = 0.071 nm) were performed on a custom-built bench by XENOCS. Aqueous solutions prepared from the **Mo_20_** crystals were loaded into 2 mm quartz capillaries. The scattered beam was recorded using a large online scanner detector (diameter: 345 mm, MAR Research). Collimation was achieved using a 12:∞ multilayer Xenocs mirror (optimized for Mo radiation) coupled with two sets of scatterless FORVIS slits, providing a 0.8 × 0.8 mm X-ray beam at the sample position. The scattered intensities are presented as a function of the scattering vector magnitude, q = [(4π)/λ]sin(θ/2), where λ is the incident radiation wavelength and θ is the scattering angle. Standard corrections for background subtraction (including empty capillary and detector noise) were applied. The experimental resolution was Δq/q = 0.05. Silver behenate in a sealed capillary was used as the calibration standard for the scattering vector. All spectra were corrected by subtracting signals from the empty capillary, and the final scattering intensities are given in arbitrary units. All experiments were conducted at 22 ± 2 °C.

**Cloud point determination.** Cloud point measurements were performed in a 1 mL cuvette under magnetic stirring. The cuvette was immersed in a water bath with controlled heating. The cloud point of octyl tetraethylene glycol ether (C8E4) at a concentration of 60 mM in water was determined to be 42 °C, in agreement with the literature. **Mo_20_** solutions were prepared in 60 mM C8E4 to maintain a constant detergent concentration. Cloud point variations were detected by simple visual inspection upon heating-cooling cycles, as some **Mo_20_**-containing solutions transitioned from transparent to cloudy upon heating, and returned to a clear state upon cooling. The thermometer used had an accuracy of ± 0.5 °C. Octyl tetraethylene glycol ether (CAS: 19327-39-0) was purchased from Sigma-Aldrich. The cloud point evolution was measured for concentration of **Mo_20_** up to 30 mM.

**X-ray Crystallography.** Single crystal X-ray diffraction (XRD) studies on Na_5_(NH_4_)_7_[Mo^VI^_20_O_60_{AsO_2_(CH_3_)_2_}_12_]·40H_2_O (**Na-NH_4_-Mo_20_**) and the same compound recrystallized from the NMR solution (abbreviated as **Na-NH_4_-Mo_20_-rec**) were performed using a Rigaku Oxford Diffraction XtaLAB Synergy diffractometer with Cu-Kα radiation (λ = 1.54184 Å) and a mirror monochromator. Data for the all-sodium salt Na_14_[((CH_3_)_2_AsO_2_)_2_⊂Mo^VI^_20_O_60_{AsO_2_(CH_3_)_2_}_12_]·26H_2_O (**Na-Mo_20_**) was recorded using Mo-Kα radiation (λ = 0.71073 Å). In all cases, a properly shaped single crystal was used for the measurement under N_2_ cooling at 100 K. Corrections for absorption effects were applied with CrysAlisPro 1.171.43.97a. The structures were resolved by direct methods using SHELXT, and full-matrix least-squares structure refinements were performed with SHELXL-2018/3 implemented in Olex2 1.5-ac5-024.^[2-5]^ All non-hydrogen atoms were refined anisotropically. Disordered solvent/guest molecules are present in the voids of the crystal structure of all compounds. The Olex2 solvent mask routine (similar to PLATON/SQUEEZE) was used to mask out the disordered electron density. The crystallographic data and structure refinement parameters for all compounds are shown in Table S1. Cambridge Crystallographic Data files CCDC 2517434 (**Na-NH_4_-Mo_20_**), 2517435 (**Na-NH_4_-Mo_20_-rec**) and 2517436 (**Na-Mo_20_**) contain the supplementary crystallographic data for this paper. These data can be obtained free of charge from The Cambridge Crystallographic Data Center via [www.ccdc.cam.ac.uk/](http://www.ccdc.cam.ac.uk/).

**Synthetic Procedures**

**Synthesis of Na_5_(NH_4_)_7_[Mo^VI^_20_O_60_{AsO_2_(CH_3_)_2_}_12_]·40H_2_O (Na-NH_4_-Mo_20_)**

Ammonium heptamolybdate tetrahydrate, (NH_4_)_6_Mo_7_O_24_·4H_2_O (124 mg, 0.1 mmol), was added to 2 mL of a 0.5 M sodium dimethylarsinate solution at pH 7.0 (or alternatively to 1.5 mL of an aqueous pH 6.5 solution containing 0.172 g (0.8 mmol) sodium dimethylarsinate). Then this mixture was stirred and heated at 80 °C for 1 h. The obtained colorless clear solution was then cooled and kept for slow evaporation at room temperature, leading to 102 mg (yield 53.1% based on Mo) of colorless crystals of **Na-NH_4_-Mo_20_** after 5-7 days. The crystals were collected by filtration and air-dried. Elemental analysis for **Na-NH_4_-Mo_20_**, calculated: Na, 2.10; Mo, 35.0; As, 16.4; C, 5.26; H, 3.31; N, 1.79. Found: Na, 2.02; Mo, 34.4; As, 17.0; C, 5.37; H, 2.68; N, 1.37. FT-IR (2% KBr disk, cm^-1^): 3440 (br), 3192 (br), 3019 (sh), 2922 (w), 2850 (w), 1639 (s), 1407 (s), 1273 (s), 945 (sh), 895 (s), 829 (sh), 785 (s), 706 (s), 655 (w), 567 (s), 468 (m) (Figure S1).

**Synthesis of Na_14_[((CH_3_)_2_AsO_2_)_2_⊂Mo^VI^_20_O_60_{AsO_2_(CH_3_)_2_}_12_]·26H_2_O (Na-Mo_20_)**

The sodium salt of tetramolybdodimethylarsinate, Na_2_[Mo^VI^_4_O_12_(OH){AsO_2_(CH_3_)_2_}] (**Na-Mo_4_**) was prepared according to Pope’s procedure.^[1]^ Then **Na-Mo_4_** (16.6 mg, 0.02 mmol) was mixed in 0.5 mL of a 0.5 M sodium dimethylarsinate solution of pH 7.0 (or alternatively to 1 mL of an aqueous pH 6 solution containing 21.4 mg (0.1 mmol) of sodium dimethylarsinate). Then this mixture was stirred and heated at 80 °C for 20 minutes. The obtained colorless clear solution was then cooled and kept for slow evaporation at room temperature. Colorless crystals of the all-sodium salt of **Mo_20_** (**Na-Mo_20_**) were collected after 3-4 days and air-dried. Yield 61.2% (15 mg) based on **Na-Mo_4_**. Elemental analysis for **Na-Mo_20_**, calculated: Na, 5.76; Mo, 34.4; As, 18.8; C, 6.0; H, 2.5. Found: Na, 6.19; Mo, 35.7; As, 19.9; C, 6.6; H, 3.4. FT-IR (2% KBr disk, cm^-1^): 3453 (br), 2922 (w), 2844 (w), 1643 (s), 1411 (s), 12743 (s), 939 (sh), 898 (s), 817(s), 787 (s), 707 (s), 657 (w), 567 (s), 466 (w) (Figure S3).

**Computational details**

**Molecular Dynamics (MD) simulations.** To investigate the distribution of cations around the polyoxometalates in aqueous solution, we performed atomistic MD simulations with explicit solvent using GROMACS 2019.3.^[6-7]^ A modified version of the AMBER14 force field (FF), previously validated for POM aggregation in diverse environments,^[8]^ was employed. In this FF, the total potential energy is expressed as the sum of bonded interactions (bond stretching, angle bending, and dihedral torsions) and non-bonded interactions. The latter are described by pairwise additive Lennard-Jones 12-6 and electrostatic potentials, including interactions between atoms separated by more than three bonds. Parameters for the POM species were derived following the protocol of López *et al.*,^[9]^ as implemented in the topoMOx code.^[10]^ Water molecules were represented using the TIP4P-Ewald model.^[11]^ Partial atomic charges were computed at the same level of theory as the DFT calculations, using quadrupolar multipole derived charges (MDC-q) obtained from the electron density.

MD trajectories were generated under fully periodic boundary conditions in a cubic simulation box of 5.86 nm per side, corresponding to a volume of ca. 202 nm^3^. Each box contained one **Mo_20_** anion, the appropriate number of counterions (sodium or sodium+ammonium) to ensure electroneutrality, and a sufficient number of explicit water molecules to fully solvate the system. Systems containing **Mo_4_** and cacodylate species included 5 **Mo_4_** units and 7 cacodylate anions, corresponding in total to 20 molybdenum centers. A cutoff of 14 Å was applied to both van der Waals and short-range Coulombic interactions. Long-range electrostatics were treated using the particle-particle mesh Ewald (PME) method. Bond constraints involving hydrogen atoms were imposed using the LINCS algorithm.

Production simulations were carried out in the canonical (NVT) ensemble for 50 ns. Trajectory data were collected every 1 ps over the final 25 ns of each simulation. All simulations were performed at 298 K, and the temperature was controlled by a velocity-rescaling thermostat. Before the production stage, all systems were equilibrated following a multi-step protocol: 1 ns under NVT conditions, followed by 1 ns under NPT conditions to allow for box size relaxation, and a final 1 ns under NVT.

Diffusion coefficients *D_PBC_* were computed from the Stokes-Einstein relation,

$D_{PBC}=\frac{1}{2d_{\alpha}}\lim_{t\to\infty} \frac{d}{dt}\frac{1}{N}\left\langle\sum_{i=1}^{N} \left| r_{\alpha,i}\left( t \right)-r_{\alpha,i}(0) \right|^{2} \right\rangle_{t_{0}}$	∙∙∙∙∙∙1

where *d* is the dimensionality of the system, *α* denotes the Cartesian coordinate (x, y, or z), *N* is the number of atoms, *r* is the position, and *t* is the time. However, in MD simulations with periodic boundary conditions, the diffusion coefficients obtained from the Einstein relation, *D_PBC_*, exhibit a systematic dependence on the simulation box size. This finite size effect arises from long-range hydrodynamic interactions (self-interaction with periodic images) and from momentum conservation. To correct for this effect, we applied the Yeh-Hummer correction,^[12]^

$D=D_{PBC}+\frac{k_{B}T\xi}{6\pi\eta L}$	∙∙∙∙∙∙2

Where *k*_B_ is the Bolztmann constant, *L* is the length of the cubic simulation cell, η is the shear viscosity of the fluid, *T* is the temperature, and ξ = 2.837297 is a geometric constant. The corrected diffusion coefficients, *D*, therefore correspond to the infinite volume limit and can be compared more directly with experimental data.

**Density Functional Theory (DFT) calculations.** All structures, including **Mo_20_**, **Mo_4_**, the cacodylate species, and the configurations extracted from the MD simulations, were fully optimized with ADF2022^[13]^ at the GGA-PBE-D3BJ level of theory using triple-ζ + double polarization (TZ2P) Slater-type basis sets. Scalar relativistic corrections were introduced by means of ZORA. Solvent effects corresponding to liquid water were included with the COSMO model, using a dielectric constant ε = 78.39. The thermodynamics of **Mo_20_** hydrolysis were evaluated for the reaction

∙∙∙∙∙∙3

using the Gibbs free energies of the species together with the experimental absolute Gibbs free energies of H_2_O and H^+^ reported by Kelly et al.,^[14]^ namely –6.3 and –265.8 kcal mol^–1^, respectively. A standard state correction of +1.89 kcal mol^–1^ was applied to all computed free energies to account for the change from the gas-phase reference state (1 atm) to the solution-phase standard state (1 M) at 25 °C, except for water, for which a correction of +4.27 kcal mol^–1^ was used, consistent with its role as bulk solvent (55.5 M) at 25 °C.

Starting from the optimized geometries, ^1^H NMR single-point calculations were performed for both the target compounds and the reference tetramethylsilane (TMS), including spin orbit (SO) corrections and employing the GIAO formalism^[15-16]^ at the OPBE/TZ2P level, following the protocols of Pascual-Borràs *et al*.^[17-18]^ The calculated chemical shifts were obtained as

δ_cal_ = σ_ref_ – σ_x_, where σ_x_ and σ_ref_ are the isotropic magnetic shielding values for the nucleus in the target and reference molecules, respectively. Further details on the magnetic shielding tensor σ and on how chemical shifts are derived from it can be found in the literature.

**Table S1**. Crystal data and structure refinements for **Na-NH_4_-Mo_20_**, **Na-NH_4_-Mo_20_-rec** and **Na-Mo_20_**.

| Compound | **Na-NH_4_-Mo_20_** | **Na-NH_4_-Mo_20_-rec** | **Na-Mo_20_** |
| --- | --- | --- | --- |
| Empirical formula^a^ | C_24_H_72_As_12_Mo_20_Na_5_O_97_  (C_24_H_180_As_12_Mo_20_N_7_Na_5_O_124_) | C_24_H_72_As_12_Mo_20_Na_4_O_93_ | C_28_H_84_As_14_Mo_20_Na_10_O_114_ |
| Formula weight^a^ | 4845.60 (5484.46) | 4758.61 | 5442.53 |
| Temperature (K) | 100(2) | 100(2) | 100(2) |
| Radiation | Cu-K*α* | Cu-K*α* | Mo-K*α* |
| Wavelength (*λ*) | 1.54184 Å | 1.54184 Å | 0.71073 Å |
| Crystal system | Orthorhombic | Monoclinic | Tetragonal |
| Space group | *Pnma* | *P*2_1_/*m* | *P*4_2_2_1_2 |
| *a* [Å] | 31.1859(2) | 11.3818(4) | 23.8688(2) |
| *b* [Å] | 23.4677(2) | 24.4417(7) | 23.8688(2) |
| *c* [Å] | 24.35480(10) | 26.4444(6) | 15.8523(2) |
| *α* [°] | 90 | 90 | 90 |
| *β* [°] | 90 | 90.688(3) | 90 |
| *γ* [°] | 90 | 90 | 90 |
| Volume [Å^3^] | 17824.3(2) | 7356.1(4) | 9031.36(19) |
| *Z* | 4 | 2 | 2 |
| Density (calculated) [Mg/m^3^] | 1.806 | 2.148 | 2.001 |
| Absorption coefficient [mm^-1^] | 14.382 | 17.360 | 3.993 |
| *F*(000) | 9132 | 4480 | 5152 |
| Refl. used [*I* > 2*σ*(*I*)] | 17535 | 9314 | 11316 |
| Independent reflections | 19742 | 16014 | 12857 |
| *R*_int_ | 0.0453 | 0.1410 | 0.0486 |
| Refinement method | full-matrix least squares on *F*^2^ | full-matrix least squares on *F*^2^ | full-matrix least squares on *F*^2^ |
| GOF | 1.043 | 1.135 | 1.050 |
| Final *R*_1_^b^*, wR*_2_^c^ [*I*>2*σ*(*I*)] | *R*_1_ = 0.0381  *wR*_2_ = 0.1046 | *R*_1_ = 0.1053  *wR*_2_ = 0.3081 | *R*_1_ = 0.0308  *wR*_2_ = 0.0784 |
| *R*_1_^b^*, wR*_2_^c^ (all data) | *R*_1_ = 0.0424  *wR*_2_ = 0.1076 | *R*_1_ = 0.1422  *wR*_2_ = 0.3418 | *R*_1_ = 0.0398  *wR*_2_ = 0.0807 |
| CCDC number | 2517434 | 2517435 | 2517436 |

^a^ The entries in brackets are the actual formula units and weights as based on bulk elemental analysis. ^b^ *R*_1_ = Σ||F_o_| − |F_c_||/Σ|F_o_|. ^c^ *wR*_2_ = [Σw(F_o_^2^ − F_c_^2^)^2^/Σw(F_o_^2^)^2^]^1/2^

**Figure S1**. FTIR spectrum of **Na-NH_4_-Mo_20_** salt on a KBr disk.

**Figure S2**. FTIR spectrum of **Na-NH_4_-Mo_20_-rec** salt on a KBr disk.

**Figure S3**. FTIR spectrum of **Na-Mo_20_** salt on a KBr disk.

**Figure S4**. Structural representation of polyanion **Mo_20_** obtained from the **Na-NH_4_-Mo_20_** salt. Color code: Mo, cyan; O, red; As, yellow; C, black; H, white.

**Figure S5**. Structural representation of polyanion **Mo_20_** obtained from the **Na-NH_4_-Mo_20_** salt indicating the positions of some selected counter cations in close proximity to the polyanion. Color code: MoO_6_, cyan polyhedra; O, red; As, yellow; C, black; H, white; Na, orange.

**Figure S6**. Structural representation of polyanion **(caco)_2_@Mo_20_** obtained from the **Na-Mo_20_** salt, showing the two dimethylarsinate guest ions in the central cavity as well as some selected counter cations in close proximity to the polyanion. Color code: MoO_6_, cyan polyhedra; O, red; As, yellow; C, black; H, white; Na, orange.

**Figure S7**. Structural representation of polyanion **(caco)_2_@Mo_20_** obtained from the **Na-Mo_20_** salt, highlighting the non-covalent C-H∙∙∙O interactions (black dotted bonds) between the methyl groups of the two dimethylarsinate guests and oxygens of the **Mo_20_** host. Color code: Mo, cyan; O, red; As, yellow; C, black; H, white; Na, orange.

**Figure S8**. Structural representation of polyanion **Mo_20_** obtained from the recrystallized salt **Na-NH_4_-Mo_20_-rec**. Color code: MoO_6_, cyan polyhedra; O, red; As, yellow; C, black; H, white; Na, orange.

**Figure S9**. Thermogram of **Na-NH_4_-Mo_20_** salt from room temperature to 500 °C under N_2_ flow.

**Figure S10**. Thermogram of **Na-Mo_20_** salt from room temperature to 500 °C under N_2_ flow.

**Figure S11**. ^1^H NMR spectrum of fresh **Na-NH_4_-Mo_20_** salt dissolved in water, recorded at room temperature.

**Figure S12**. ^13^C NMR spectrum of fresh **Na-NH_4_-Mo_20_** salt dissolved in water, recorded at room temperature.

**Figure S13**. Comparative ^1^H NMR spectra, recorded at room temperature in water.

**Figure S14**. Comparative ^13^C NMR spectra, recorded at room temperature in water.

**Figure S15**. ^1^H NMR spectra of **Na-NH_4_-Mo_20_** and **Na-NH_4_-Mo_20_-rec** salts dissolved in water, recorded at room temperature.

**Figure S16**. ^1^H NMR spectrum of fresh **Na-Mo_20_** salt dissolved in water, recorded at room temperature.

**Figure S17**. ^13^C NMR spectrum of fresh **Na-Mo_20_** salt dissolved in water, recorded at room temperature.

**Figure S18.** ESI-mass spectrum in negative ion mode of **Na-NH_4_-Mo_20_** salt in aqueous solution; **A** experimental spectrum zoomed in around *m/z* 588 of [Mo_4_H_2_LiO_11_]^-^; **B** simulated spectrum zoomed in around *m/z* 588 of [Mo_4_H_2_LiO_11_]^-^; **C** experimental spectrum zoomed in around *m/z* 872 of [Mo_4_C_4_H_12_As_2_LiO_17_]^-^; **D** simulated spectrum zoomed in around *m/z* 872 of [Mo_4_C_4_H_12_As_2_LiO_17_]^-^.

**Figure S19.** (a) Combined RDFs for **Mo_20_**···Na^+^ (solid orange line) and **Mo_20_**···NH_4_^+^ (solid blue line), referenced to the respective COMs, with their integrations (orange and blue dashed lines). The first-peak position (1^st^), integration cutoff (*I*), both in Å, and the corresponding coordination number (*N*) are reported. (b) Two views of **Na-NH_4_-Mo_20_** highlighting internal vs external cations. Internal cavity occupants and surface-bound cations are shown explicitly. Color code: MoO_6_ octahedra, cyan polyhedra; O, red; As, yellow; C, gray; H, white; Na, orange; N, blue.

**Figure S20.** (a) Combined RDFs for cacodylate(As)···Na^+^ (solid orange line) and cacodylate(As)···NH_4_^+^ (solid blue line), referenced to the respective COMs, with their integrations (orange and blue dashed lines). The first-peak position (1^st^), integration cutoff (*I*), both in Å, and the corresponding coordination number (*N*) are reported. (b) Combined RDFs for **Mo_4_**···Na^+^ (solid orange line), **Mo_4_**···NH_4_^+^ (solid blue line), and **Mo_4_**···H_2_O (solid green line), referenced to the respective COMs, with their integrations (orange, blue and green dashed lines). The first-peak position (1^st^), integration cutoff (*I*), both in Å, and the corresponding coordination number (*N*) are reported.

**References**

[1] K. M. Barkigia, L. M. Rajković-Blazer, M. T. Pope, E. Prince, C. O. Quicksall, *Inorg. Chem.* **1980**, *19*, 2531-2537.

[2] G. M. Sheldrick, *Acta Crystallogr. Sect. A Found. Crystallogr.* **2015**, *71*, 3-8.

[3] O. V. Dolomanov, L. J. Bourhis, R. J. Gildea, J. A. K. Howard, H. Puschmann, *J. Appl. Crystallogr.* **2009**, *42*, 339-341.

[4] G. M. Sheldrick, *Acta Crystallogr. Sect. C Struct. Chem.* **2015**, *71*, 3-8.

[5] L. J. Bourhis, O. V. Dolomanov, R. J. Gildea, J. A. K. Howard, H. Puschmann, *Acta Crystallogr. Sect. A Found. Crystallogr.* **2015**, *71*, 59-75.

[6] B. Hess, C. Kutzner, D. van der Spoel, E. Lindahl, *J. Chem. Theory Comput.* **2008**, *4*, 435-447.

[7] J. Wang, P. Cieplak, P. A. Kollman, *J. Comput. Chem*. **2000**, *21*, 1049-1074.

[8] A. Chaumont, G. Wipff, *J. Phys. Chem. C* **2009**, *113*, 18233-18243.

[9] X. López, C. Nieto-Draghi, C. Bo, J. B. Avalos, J. M. Poblet, *J. Phys. Chem. A* **2005**, *109*, 1216-1222.

[10] A. Masip-Sánchez, topoMOx: Topologies for Metal Oxides. **2024**; <https://github.com/qcgurv/topoMOx>

[11] H. W. Horn, W. C. Swope, J. W. Pitera, J. D. Madura, T. J. Dick, G. L. Hura, T. Head-Gordon, *J. Chem. Phys.* **2004**, *120*, 9665-9678.

[12] I.-C. Yeh, G. Hummer, *J. Phys. Chem. B* **2004,** *108,* 15873-15879.

[13] G. Te Velde, F. M. Bickelhaupt, E. J. Baerends, C. Fonseca Guerra, S. J. A. van Gisbergen, J. G. Snijders, T. Ziegler, *J. Comput. Chem*. [**2001**, *22*, 931-967.](https://doi.org/10.1002/jcc.1056)

[14] C. P. Kelly, C. J. Cramer, D. G. Truhlar, *J. Phys. Chem. B* **2006,** *110,* 16066-16081.

[15] R. Ditchfield, *Mol. Phys.* **1974**, *27*, 789-807.

[16] K. Wolinski, J. F. Hinton, P. Pulay, *J. Am. Chem. Soc.* **1990**, *112*, 8251-8260.

[17] M. Pascual-Borràs, X. López, A. Rodríguez-Fortea, R. J. Errington, J. M. Poblet, *Chem. Sci.* **2014**, *5*, 2031-2042.

[18] M. Pascual-Borràs, X. López, J. M. Poblet, *Phys. Chem. Chem. Phys.* **2015**, *17*, 8723-8731.
